# Supplementary material for: Identifying High-Risk Tumors within AJCC Stage IB–III Melanomas Using a Seven-Marker Immunohistochemical Signature
Source: Cancers (Basel). 2021 Jun 10;13(12):2902. doi: 10.3390/cancers13122902 (PMC8229951; doi:10.3390/cancers13122902)
Supplement: Supplementary file 1 [file cancers-13-02902-s001.zip › cancers-1247549-supplementary/cancers-1247549-supplementary for XML/Supplement Figures S1-S5 .pptx]

## Slide 1
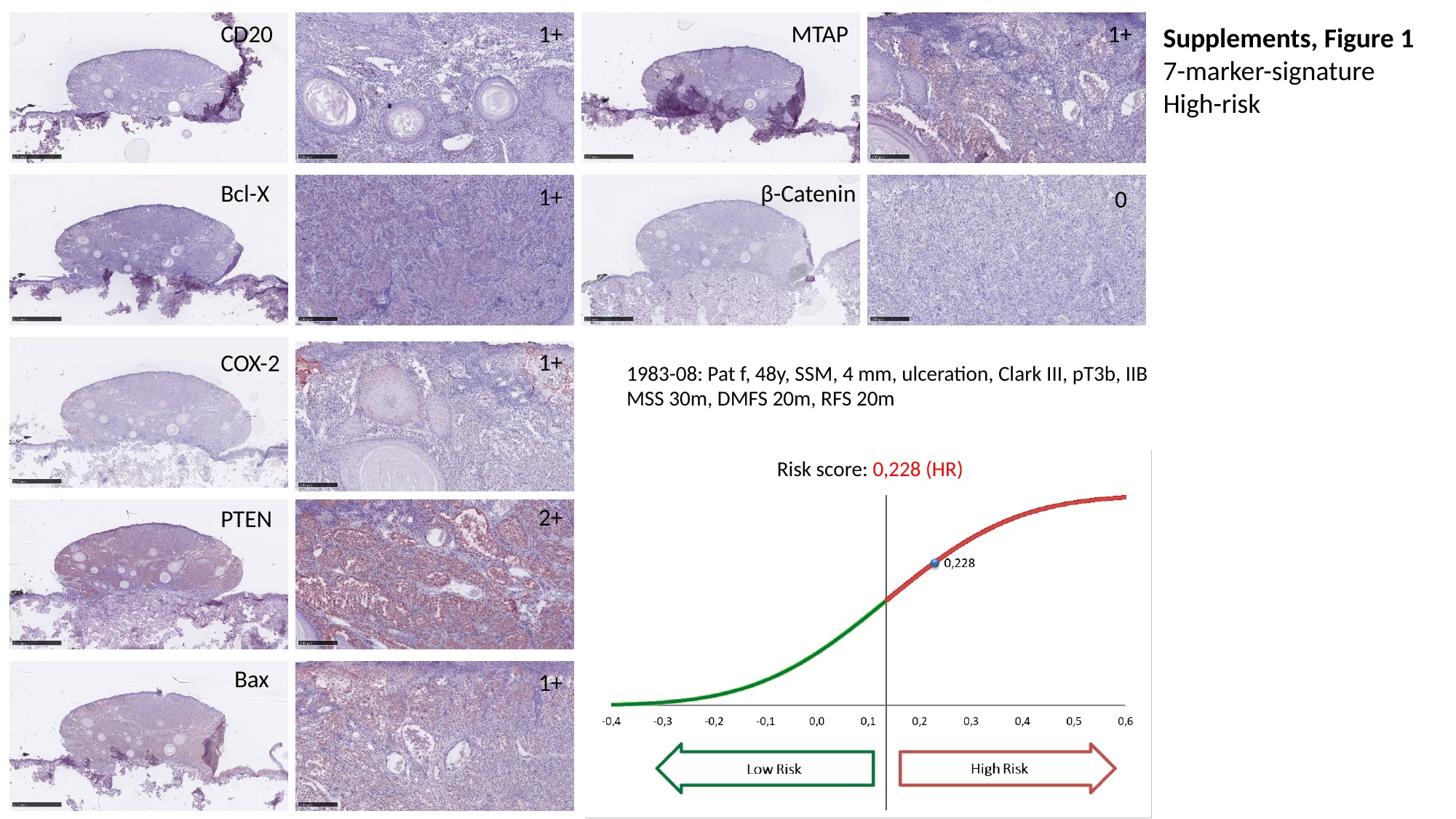

1+
MTAP
CD20
1+
Supplements, Figure 1
7-marker-signature
High-risk
Bcl-X
β-Catenin
1+
0
1+
COX-2
1983-08: Pat f, 48y, SSM, 4 mm, ulceration, Clark III, pT3b, IIB
MSS 30m, DMFS 20m, RFS 20m
Risk score: 0,228 (HR)
2+
PTEN
Bax
1+

## Slide 2
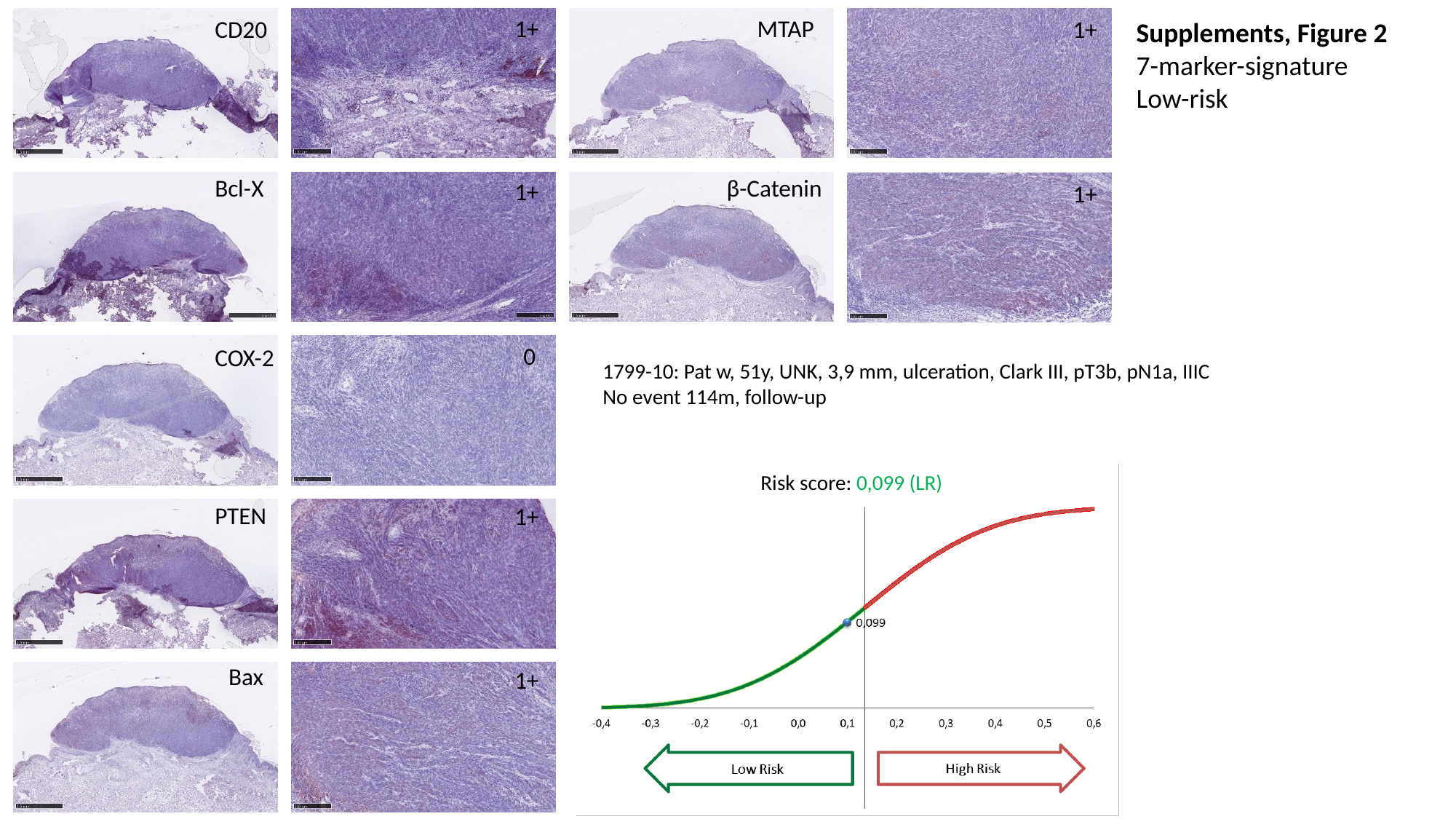

1+
MTAP
CD20
1+
Supplements, Figure 2
7-marker-signature
Low-risk
Bcl-X
β-Catenin
1+
1+
0
COX-2
1799-10: Pat w, 51y, UNK, 3,9 mm, ulceration, Clark III, pT3b, pN1a, IIIC
No event 114m, follow-up
Risk score: 0,099 (LR)
PTEN
1+
Bax
1+

## Slide 3
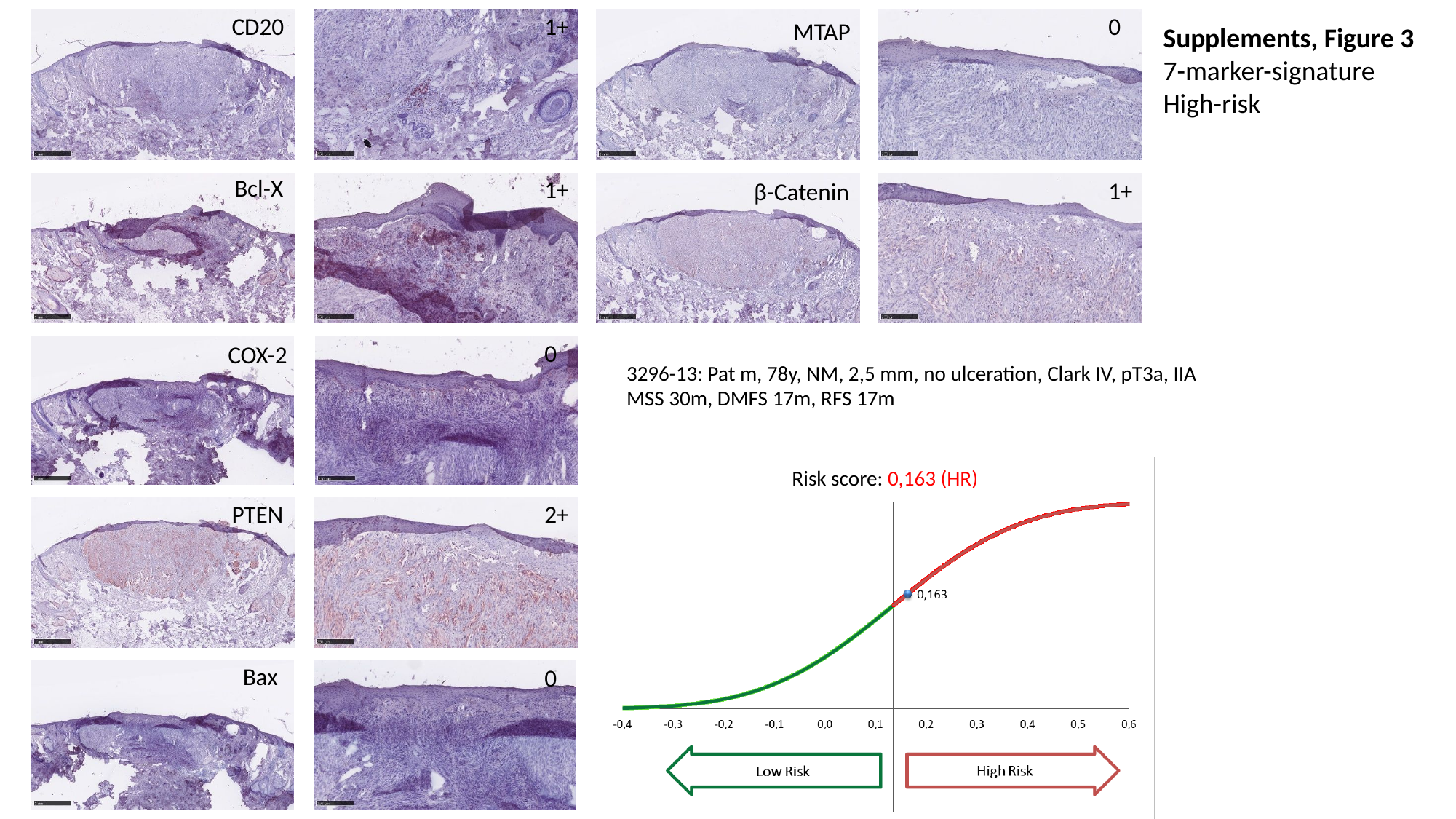

1+
CD20
0
MTAP
Supplements, Figure 3
7-marker-signature
High-risk
Bcl-X
1+
1+
β-Catenin
0
COX-2
3296-13: Pat m, 78y, NM, 2,5 mm, no ulceration, Clark IV, pT3a, IIA
MSS 30m, DMFS 17m, RFS 17m
Risk score: 0,163 (HR)
2+
PTEN
Bax
0

## Slide 4
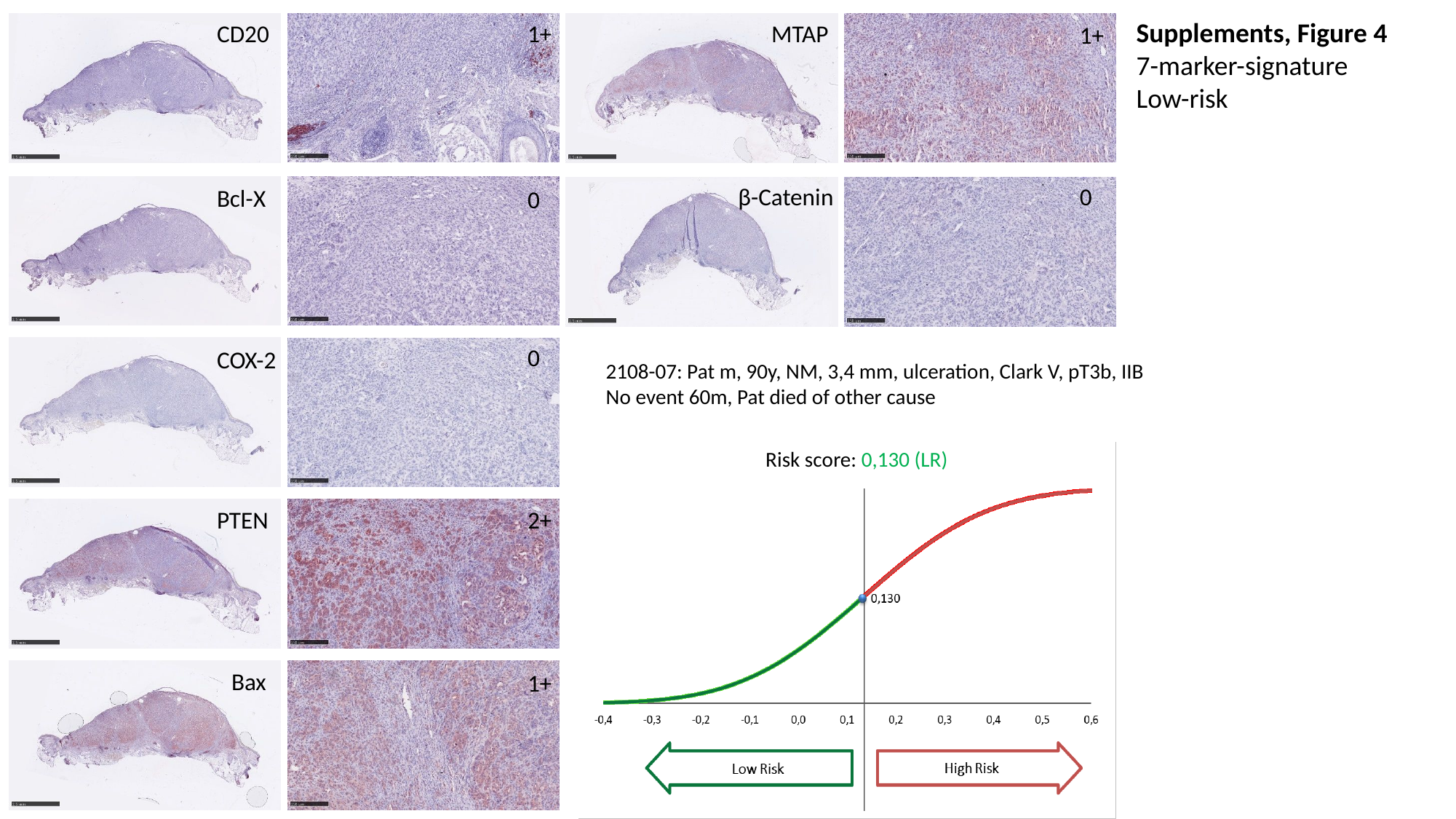

Supplements, Figure 4
7-marker-signature
Low-risk
CD20
1+
MTAP
1+
0
β-Catenin
Bcl-X
0
0
COX-2
2108-07: Pat m, 90y, NM, 3,4 mm, ulceration, Clark V, pT3b, IIB
No event 60m, Pat died of other cause
Risk score: 0,130 (LR)
PTEN
2+
Bax
1+

## Slide 5
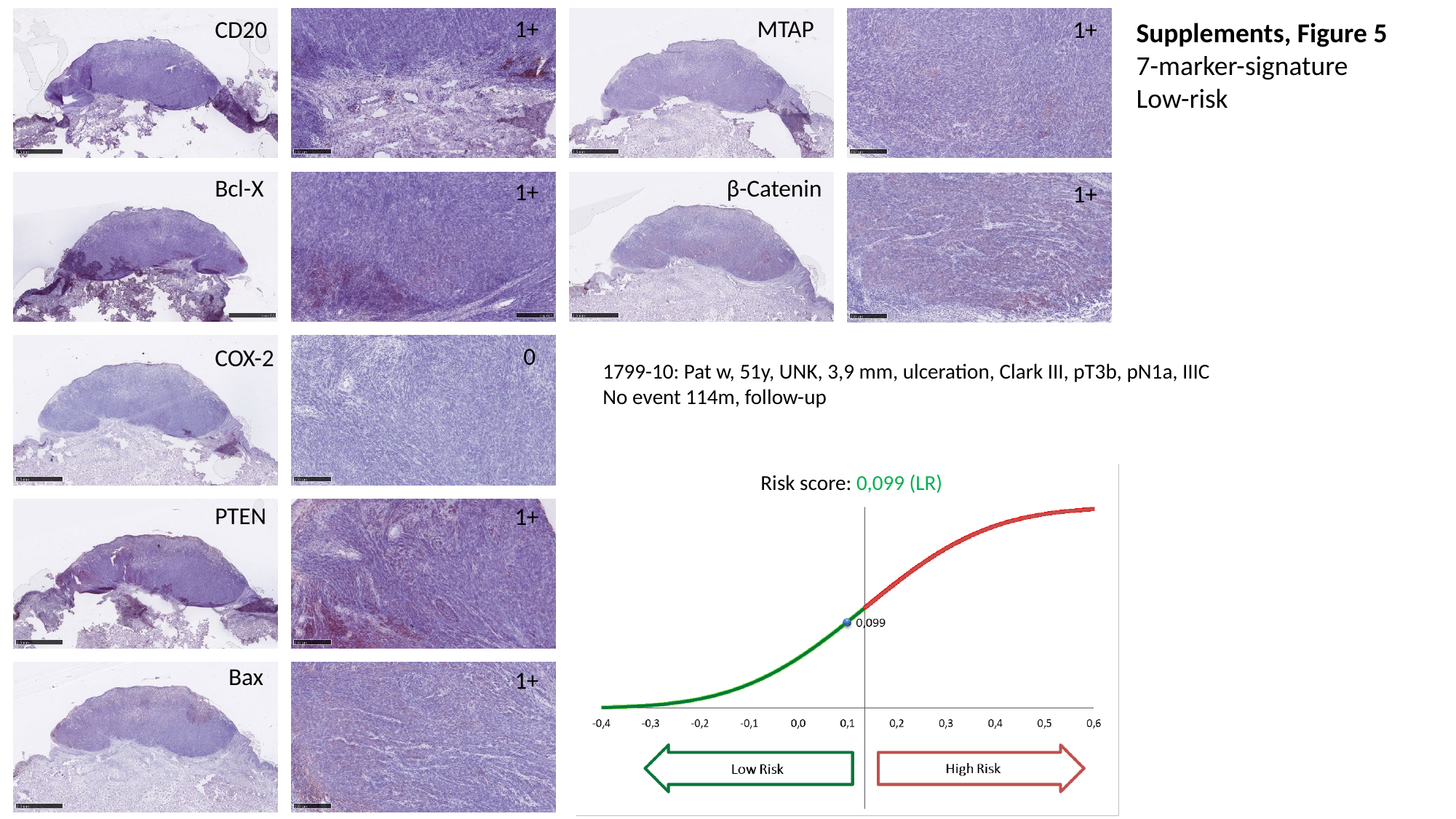

1+
MTAP
CD20
1+
Supplements, Figure 5
7-marker-signature
Low-risk
Bcl-X
β-Catenin
1+
1+
0
COX-2
1799-10: Pat w, 51y, UNK, 3,9 mm, ulceration, Clark III, pT3b, pN1a, IIIC
No event 114m, follow-up
Risk score: 0,099 (LR)
PTEN
1+
Bax
1+
