# Supplementary material for: Identifying High-Risk Tumors within AJCC Stage IB–III Melanomas Using a Seven-Marker Immunohistochemical Signature
Source: Cancers (Basel). 2021 Jun 10;13(12):2902. doi: 10.3390/cancers13122902 (PMC8229951; doi:10.3390/cancers13122902)
Supplement: Supplementary file 1 [file cancers-13-02902-s001.zip › cancers-1247549-supplementary/cancers-1247549-supplementary for XML/Supplement Figures S6-S8.pptx]

## Slide 1
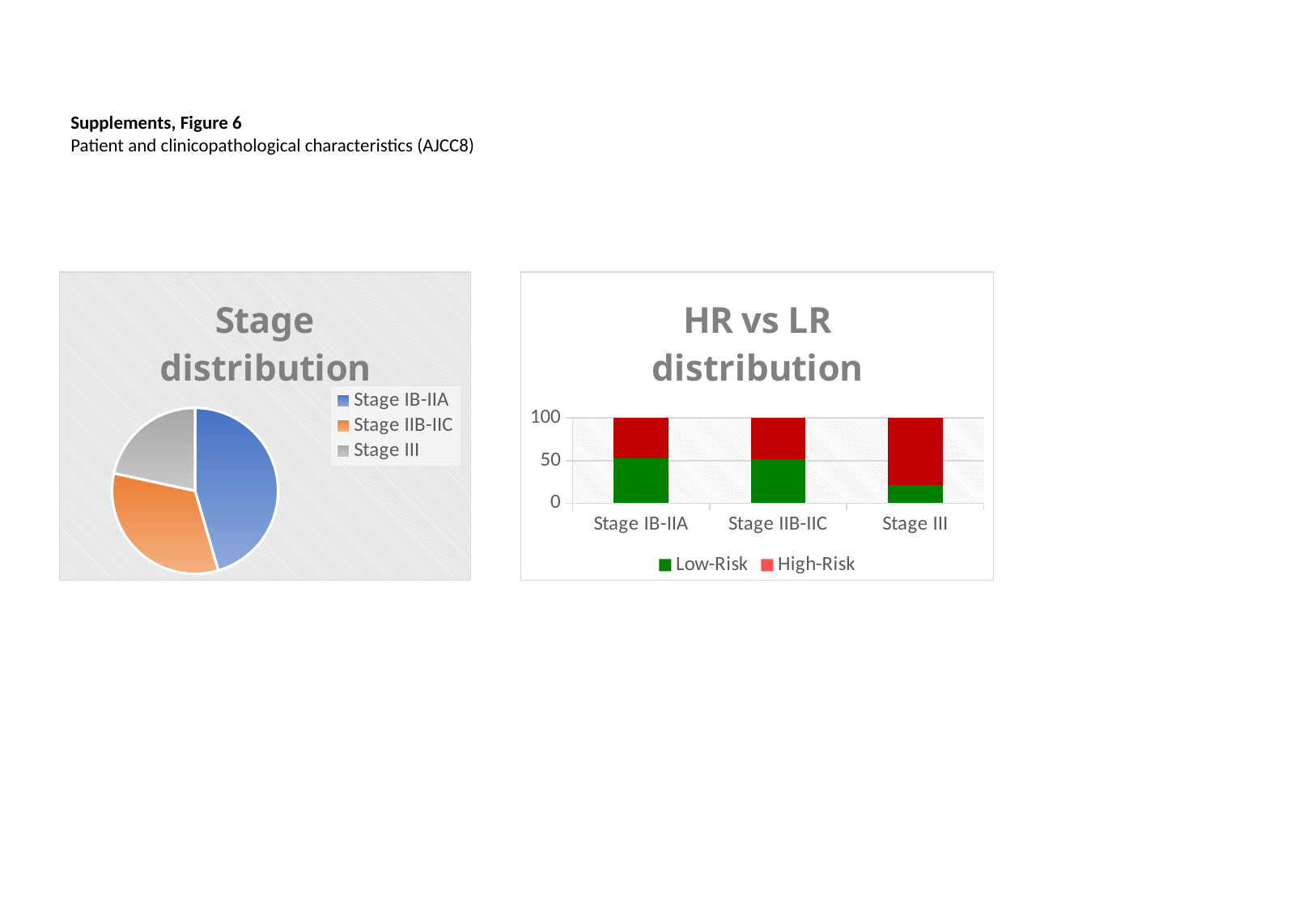

Supplements, Figure 6
Patient and clinicopathological characteristics (AJCC8)
### Chart:
| Category | Stage distribution |
|---|---|
| Stage IB-IIA | 45.5 |
| Stage IIB-IIC | 32.9 |
| Stage III | 21.6 |
### Chart: HR vs LR distribution
| Category | Low-Risk | High-Risk |
|---|---|---|
| Stage IB-IIA | 52.5 | 47.5 |
| Stage IIB-IIC | 51.7 | 48.2 |
| Stage III | 21.1 | 78.9 |

## Slide 2
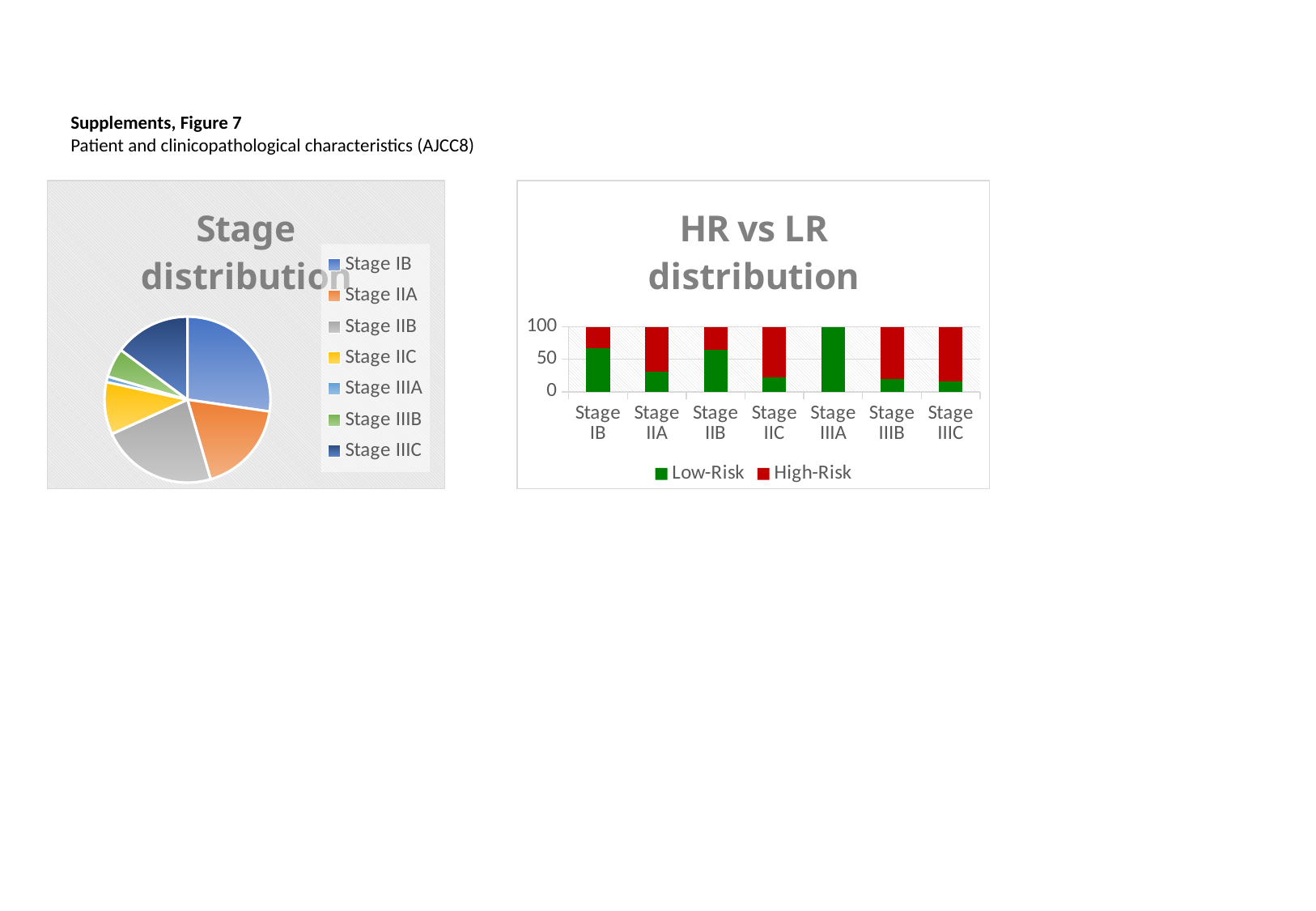

Supplements, Figure 7
Patient and clinicopathological characteristics (AJCC8)
### Chart:
| Category | Stage distribution |
|---|---|
| Stage IB | 27.3 |
| Stage IIA | 18.2 |
| Stage IIB | 22.7 |
| Stage IIC | 10.2 |
| Stage IIIA | 1.1 |
| Stage IIIB | 5.7 |
| Stage IIIC | 14.8 |
### Chart: HR vs LR distribution
| Category | Low-Risk | High-Risk |
|---|---|---|
| Stage IB | 66.7 | 33.3 |
| Stage IIA | 31.3 | 68.8 |
| Stage IIB | 65.0 | 35.0 |
| Stage IIC | 22.2 | 77.8 |
| Stage IIIA | 100.0 | 0.0 |
| Stage IIIB | 20.0 | 80.0 |
| Stage IIIC | 15.4 | 84.6 |

## Slide 3
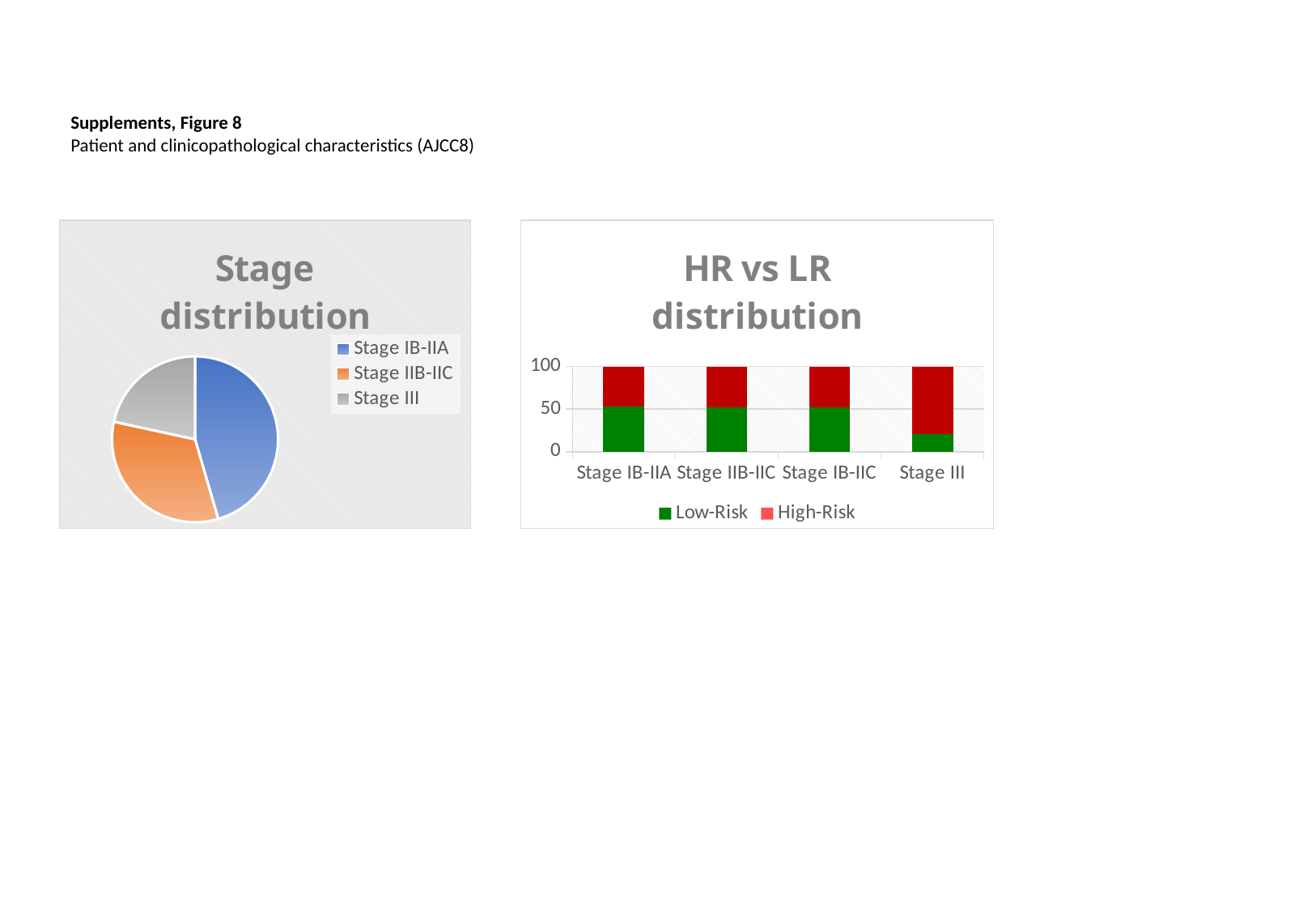

Supplements, Figure 8
Patient and clinicopathological characteristics (AJCC8)
### Chart:
| Category | Stage distribution |
|---|---|
| Stage IB-IIA | 45.5 |
| Stage IIB-IIC | 32.9 |
| Stage III | 21.6 |
### Chart: HR vs LR distribution
| Category | Low-Risk | High-Risk |
|---|---|---|
| Stage IB-IIA | 52.5 | 47.5 |
| Stage IIB-IIC | 51.7 | 48.2 |
| Stage IB-IIC | 52.2 | 47.8 |
| Stage III | 21.1 | 78.9 |
