# Supplementary material for: Identifying High-Risk Tumors within AJCC Stage IB–III Melanomas Using a Seven-Marker Immunohistochemical Signature
Source: Cancers (Basel). 2021 Jun 10;13(12):2902. doi: 10.3390/cancers13122902 (PMC8229951; doi:10.3390/cancers13122902)
Supplement: Supplementary file 1 [file cancers-13-02902-s001.zip › cancers-1247549-supplementary/cancers-1247549-supplementary for XML/Supplement Table S1.pptx]

## Slide 1
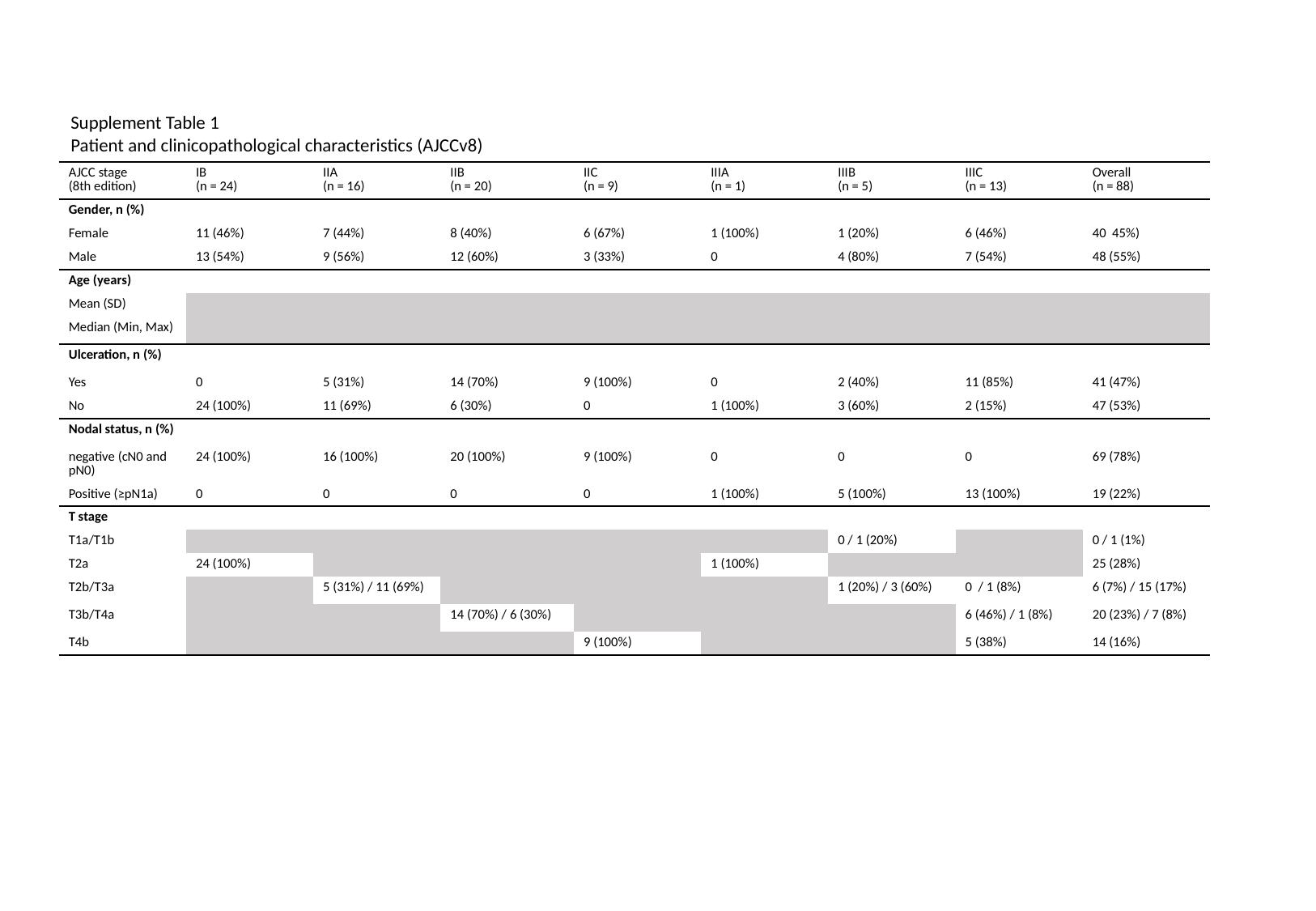

Supplement Table 1
Patient and clinicopathological characteristics (AJCCv8)
| AJCC stage (8th edition) | IB (n = 24) | IIA (n = 16) | IIB (n = 20) | IIC (n = 9) | IIIA (n = 1) | IIIB (n = 5) | IIIC (n = 13) | Overall (n = 88) |
| --- | --- | --- | --- | --- | --- | --- | --- | --- |
| Gender, n (%) | | | | | | | | |
| Female | 11 (46%) | 7 (44%) | 8 (40%) | 6 (67%) | 1 (100%) | 1 (20%) | 6 (46%) | 40 45%) |
| Male | 13 (54%) | 9 (56%) | 12 (60%) | 3 (33%) | 0 | 4 (80%) | 7 (54%) | 48 (55%) |
| Age (years) | | | | | | | | |
| Mean (SD) | | | | | | | | |
| Median (Min, Max) | | | | | | | | |
| Ulceration, n (%) | | | | | | | | |
| Yes | 0 | 5 (31%) | 14 (70%) | 9 (100%) | 0 | 2 (40%) | 11 (85%) | 41 (47%) |
| No | 24 (100%) | 11 (69%) | 6 (30%) | 0 | 1 (100%) | 3 (60%) | 2 (15%) | 47 (53%) |
| Nodal status, n (%) | | | | | | | | |
| negative (cN0 and pN0) | 24 (100%) | 16 (100%) | 20 (100%) | 9 (100%) | 0 | 0 | 0 | 69 (78%) |
| Positive (≥pN1a) | 0 | 0 | 0 | 0 | 1 (100%) | 5 (100%) | 13 (100%) | 19 (22%) |
| T stage | | | | | | | | |
| T1a/T1b | | | | | | 0 / 1 (20%) | | 0 / 1 (1%) |
| T2a | 24 (100%) | | | | 1 (100%) | | | 25 (28%) |
| T2b/T3a | | 5 (31%) / 11 (69%) | | | | 1 (20%) / 3 (60%) | 0 / 1 (8%) | 6 (7%) / 15 (17%) |
| T3b/T4a | | | 14 (70%) / 6 (30%) | | | | 6 (46%) / 1 (8%) | 20 (23%) / 7 (8%) |
| T4b | | | | 9 (100%) | | | 5 (38%) | 14 (16%) |
