# Supplementary material for: Identifying High-Risk Tumors within AJCC Stage IB–III Melanomas Using a Seven-Marker Immunohistochemical Signature
Source: Cancers (Basel). 2021 Jun 10;13(12):2902. doi: 10.3390/cancers13122902 (PMC8229951; doi:10.3390/cancers13122902)
Supplement: Supplementary file 1 [file cancers-13-02902-s001.zip › cancers-1247549-supplementary/cancers-1247549-supplementary for XML/Supplement Table S2.pptx]

## Slide 1
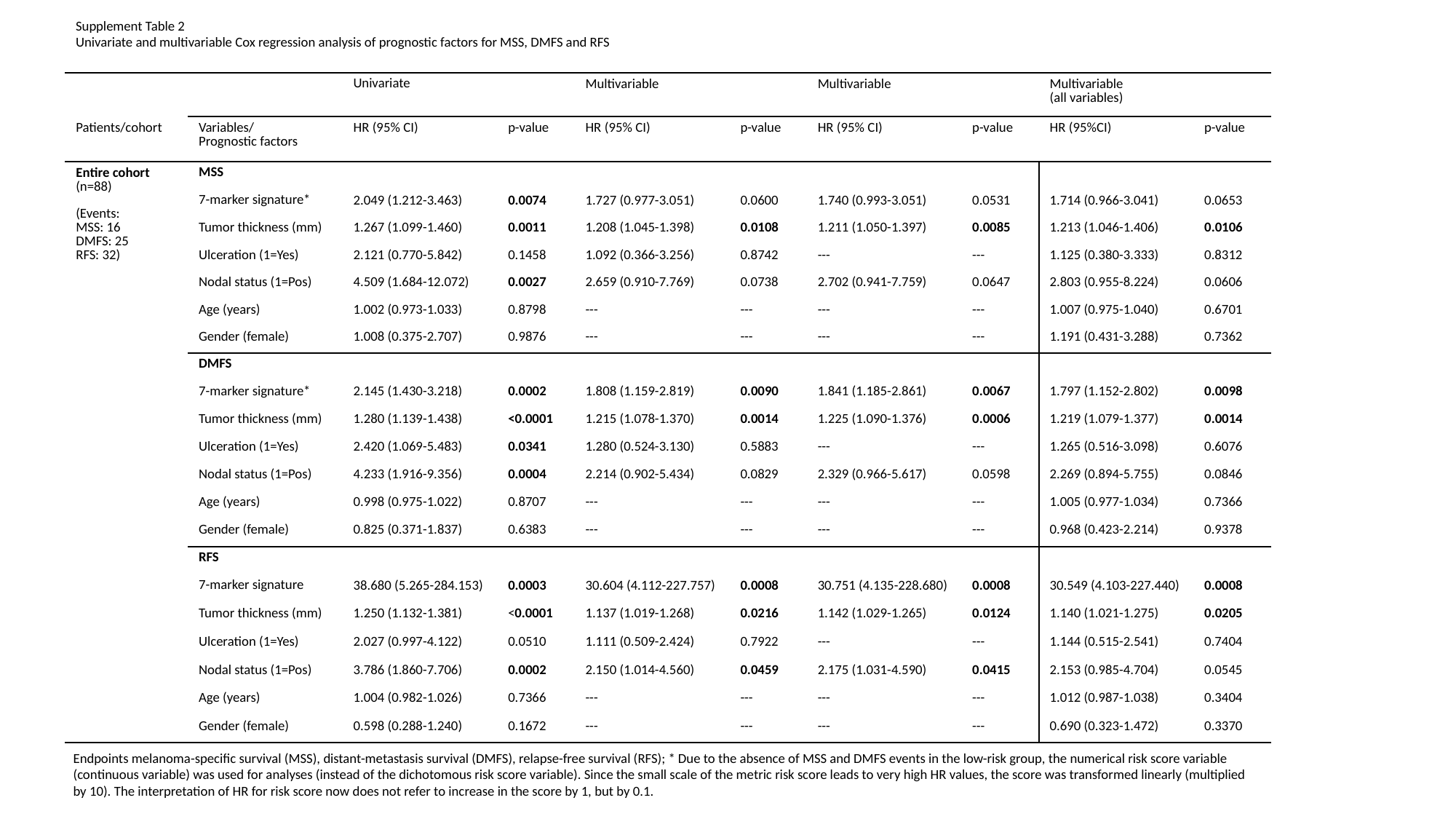

Supplement Table 2
Univariate and multivariable Cox regression analysis of prognostic factors for MSS, DMFS and RFS
| | | Univariate | | Multivariable | | Multivariable | | Multivariable (all variables) | |
| --- | --- | --- | --- | --- | --- | --- | --- | --- | --- |
| Patients/cohort | Variables/ Prognostic factors | HR (95% CI) | p-value | HR (95% CI) | p-value | HR (95% CI) | p-value | HR (95%CI) | p-value |
| Entire cohort (n=88) (Events: MSS: 16 DMFS: 25 RFS: 32) | MSS | | | | | | | | |
| Entire cohort (n=88) | 7-marker signature\* | 2.049 (1.212-3.463) | 0.0074 | 1.727 (0.977-3.051) | 0.0600 | 1.740 (0.993-3.051) | 0.0531 | 1.714 (0.966-3.041) | 0.0653 |
| | Tumor thickness (mm) | 1.267 (1.099-1.460) | 0.0011 | 1.208 (1.045-1.398) | 0.0108 | 1.211 (1.050-1.397) | 0.0085 | 1.213 (1.046-1.406) | 0.0106 |
| (Events: RFS: XX DMFS: YY MSS: ZZ) | Ulceration (1=Yes) | 2.121 (0.770-5.842) | 0.1458 | 1.092 (0.366-3.256) | 0.8742 | --- | --- | 1.125 (0.380-3.333) | 0.8312 |
| | Nodal status (1=Pos) | 4.509 (1.684-12.072) | 0.0027 | 2.659 (0.910-7.769) | 0.0738 | 2.702 (0.941-7.759) | 0.0647 | 2.803 (0.955-8.224) | 0.0606 |
| | Age (years) | 1.002 (0.973-1.033) | 0.8798 | --- | --- | --- | --- | 1.007 (0.975-1.040) | 0.6701 |
| | Gender (female) | 1.008 (0.375-2.707) | 0.9876 | --- | --- | --- | --- | 1.191 (0.431-3.288) | 0.7362 |
| | DMFS | | | | | | | | |
| | 7-marker signature\* | 2.145 (1.430-3.218) | 0.0002 | 1.808 (1.159-2.819) | 0.0090 | 1.841 (1.185-2.861) | 0.0067 | 1.797 (1.152-2.802) | 0.0098 |
| | Tumor thickness (mm) | 1.280 (1.139-1.438) | <0.0001 | 1.215 (1.078-1.370) | 0.0014 | 1.225 (1.090-1.376) | 0.0006 | 1.219 (1.079-1.377) | 0.0014 |
| | Ulceration (1=Yes) | 2.420 (1.069-5.483) | 0.0341 | 1.280 (0.524-3.130) | 0.5883 | --- | --- | 1.265 (0.516-3.098) | 0.6076 |
| | Nodal status (1=Pos) | 4.233 (1.916-9.356) | 0.0004 | 2.214 (0.902-5.434) | 0.0829 | 2.329 (0.966-5.617) | 0.0598 | 2.269 (0.894-5.755) | 0.0846 |
| | Age (years) | 0.998 (0.975-1.022) | 0.8707 | --- | --- | --- | --- | 1.005 (0.977-1.034) | 0.7366 |
| | Gender (female) | 0.825 (0.371-1.837) | 0.6383 | --- | --- | --- | --- | 0.968 (0.423-2.214) | 0.9378 |
| | RFS | | | | | | | | |
| | 7-marker signature | 38.680 (5.265-284.153) | 0.0003 | 30.604 (4.112-227.757) | 0.0008 | 30.751 (4.135-228.680) | 0.0008 | 30.549 (4.103-227.440) | 0.0008 |
| | Tumor thickness (mm) | 1.250 (1.132-1.381) | <0.0001 | 1.137 (1.019-1.268) | 0.0216 | 1.142 (1.029-1.265) | 0.0124 | 1.140 (1.021-1.275) | 0.0205 |
| | Ulceration (1=Yes) | 2.027 (0.997-4.122) | 0.0510 | 1.111 (0.509-2.424) | 0.7922 | --- | --- | 1.144 (0.515-2.541) | 0.7404 |
| | Nodal status (1=Pos) | 3.786 (1.860-7.706) | 0.0002 | 2.150 (1.014-4.560) | 0.0459 | 2.175 (1.031-4.590) | 0.0415 | 2.153 (0.985-4.704) | 0.0545 |
| | Age (years) | 1.004 (0.982-1.026) | 0.7366 | --- | --- | --- | --- | 1.012 (0.987-1.038) | 0.3404 |
| | Gender (female) | 0.598 (0.288-1.240) | 0.1672 | --- | --- | --- | --- | 0.690 (0.323-1.472) | 0.3370 |
Endpoints melanoma-specific survival (MSS), distant-metastasis survival (DMFS), relapse-free survival (RFS); * Due to the absence of MSS and DMFS events in the low-risk group, the numerical risk score variable (continuous variable) was used for analyses (instead of the dichotomous risk score variable). Since the small scale of the metric risk score leads to very high HR values, the score was transformed linearly (multiplied by 10). The interpretation of HR for risk score now does not refer to increase in the score by 1, but by 0.1.
