# Supplementary material for: Identifying High-Risk Tumors within AJCC Stage IB–III Melanomas Using a Seven-Marker Immunohistochemical Signature
Source: Cancers (Basel). 2021 Jun 10;13(12):2902. doi: 10.3390/cancers13122902 (PMC8229951; doi:10.3390/cancers13122902)
Supplement: Supplementary file 1 [file cancers-13-02902-s001.zip › cancers-1247549-supplementary/cancers-1247549-supplementary for XML/Supplement Table S3.pptx]

## Slide 1
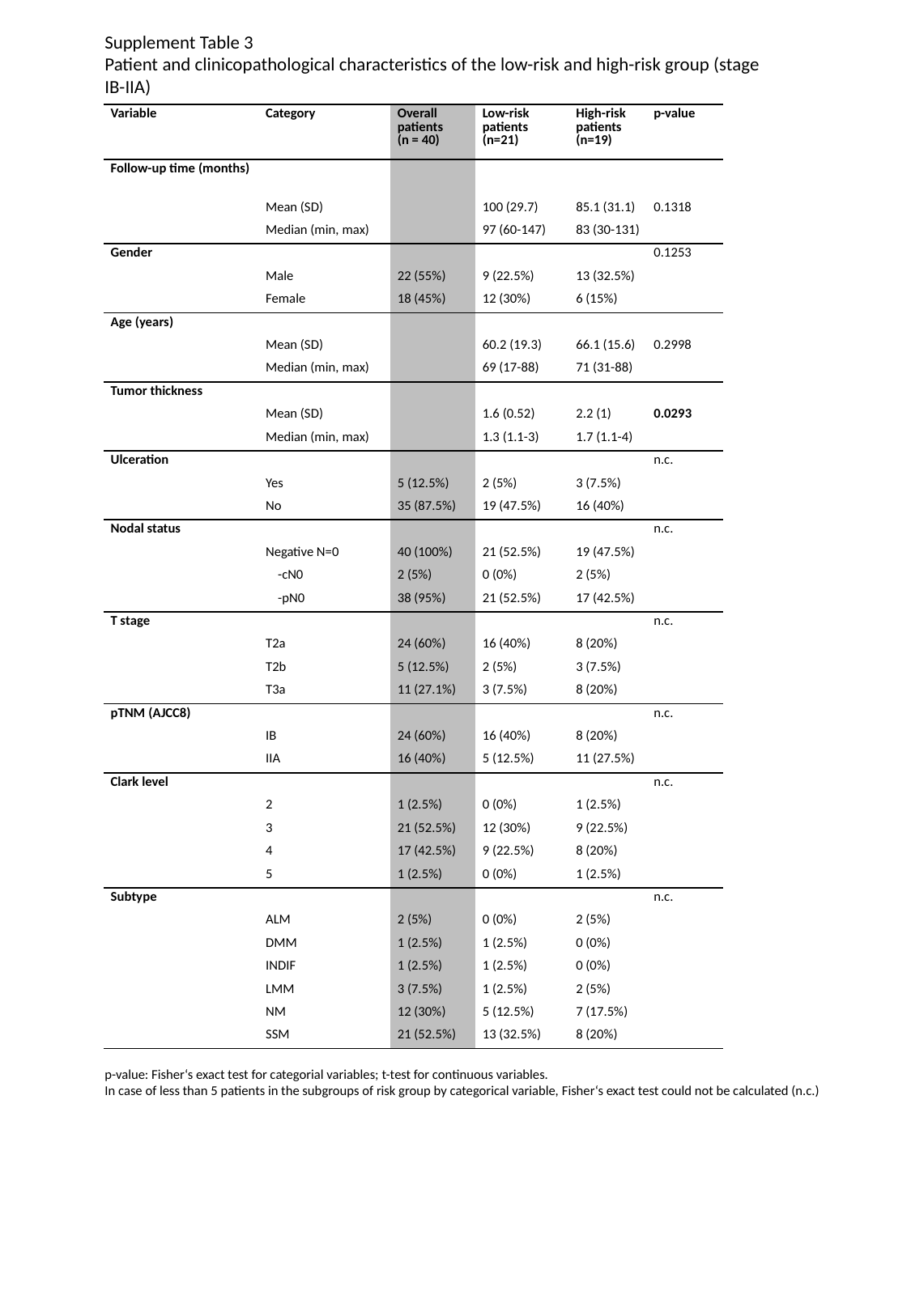

Supplement Table 3
Patient and clinicopathological characteristics of the low-risk and high-risk group (stage IB-IIA)
| Variable | Category | Overall patients (n = 40) | Low-risk patients (n=21) | High-risk patients (n=19) | p-value |
| --- | --- | --- | --- | --- | --- |
| Follow-up time (months) | | | | | |
| | Mean (SD) | | 100 (29.7) | 85.1 (31.1) | 0.1318 |
| | Median (min, max) | | 97 (60-147) | 83 (30-131) | |
| Gender | | | | | 0.1253 |
| | Male | 22 (55%) | 9 (22.5%) | 13 (32.5%) | |
| | Female | 18 (45%) | 12 (30%) | 6 (15%) | |
| Age (years) | | | | | |
| | Mean (SD) | | 60.2 (19.3) | 66.1 (15.6) | 0.2998 |
| | Median (min, max) | | 69 (17-88) | 71 (31-88) | |
| Tumor thickness | | | | | |
| | Mean (SD) | | 1.6 (0.52) | 2.2 (1) | 0.0293 |
| | Median (min, max) | | 1.3 (1.1-3) | 1.7 (1.1-4) | |
| Ulceration | | | | | n.c. |
| | Yes | 5 (12.5%) | 2 (5%) | 3 (7.5%) | |
| | No | 35 (87.5%) | 19 (47.5%) | 16 (40%) | |
| Nodal status | | | | | n.c. |
| | Negative N=0 | 40 (100%) | 21 (52.5%) | 19 (47.5%) | |
| | -cN0 | 2 (5%) | 0 (0%) | 2 (5%) | |
| | -pN0 | 38 (95%) | 21 (52.5%) | 17 (42.5%) | |
| T stage | | | | | n.c. |
| | T2a | 24 (60%) | 16 (40%) | 8 (20%) | |
| | T2b | 5 (12.5%) | 2 (5%) | 3 (7.5%) | |
| | T3a | 11 (27.1%) | 3 (7.5%) | 8 (20%) | |
| pTNM (AJCC8) | | | | | n.c. |
| | IB | 24 (60%) | 16 (40%) | 8 (20%) | |
| | IIA | 16 (40%) | 5 (12.5%) | 11 (27.5%) | |
| Clark level | | | | | n.c. |
| | 2 | 1 (2.5%) | 0 (0%) | 1 (2.5%) | |
| | 3 | 21 (52.5%) | 12 (30%) | 9 (22.5%) | |
| | 4 | 17 (42.5%) | 9 (22.5%) | 8 (20%) | |
| | 5 | 1 (2.5%) | 0 (0%) | 1 (2.5%) | |
| Subtype | | | | | n.c. |
| | ALM | 2 (5%) | 0 (0%) | 2 (5%) | |
| | DMM | 1 (2.5%) | 1 (2.5%) | 0 (0%) | |
| | INDIF | 1 (2.5%) | 1 (2.5%) | 0 (0%) | |
| | LMM | 3 (7.5%) | 1 (2.5%) | 2 (5%) | |
| | NM | 12 (30%) | 5 (12.5%) | 7 (17.5%) | |
| | SSM | 21 (52.5%) | 13 (32.5%) | 8 (20%) | |
p-value: Fisher‘s exact test for categorial variables; t-test for continuous variables.
In case of less than 5 patients in the subgroups of risk group by categorical variable, Fisher‘s exact test could not be calculated (n.c.)
