# Supplementary material for: Identifying High-Risk Tumors within AJCC Stage IB–III Melanomas Using a Seven-Marker Immunohistochemical Signature
Source: Cancers (Basel). 2021 Jun 10;13(12):2902. doi: 10.3390/cancers13122902 (PMC8229951; doi:10.3390/cancers13122902)
Supplement: Supplementary file 1 [file cancers-13-02902-s001.zip › cancers-1247549-supplementary/cancers-1247549-supplementary for XML/Supplement Table S4.pptx]

## Slide 1
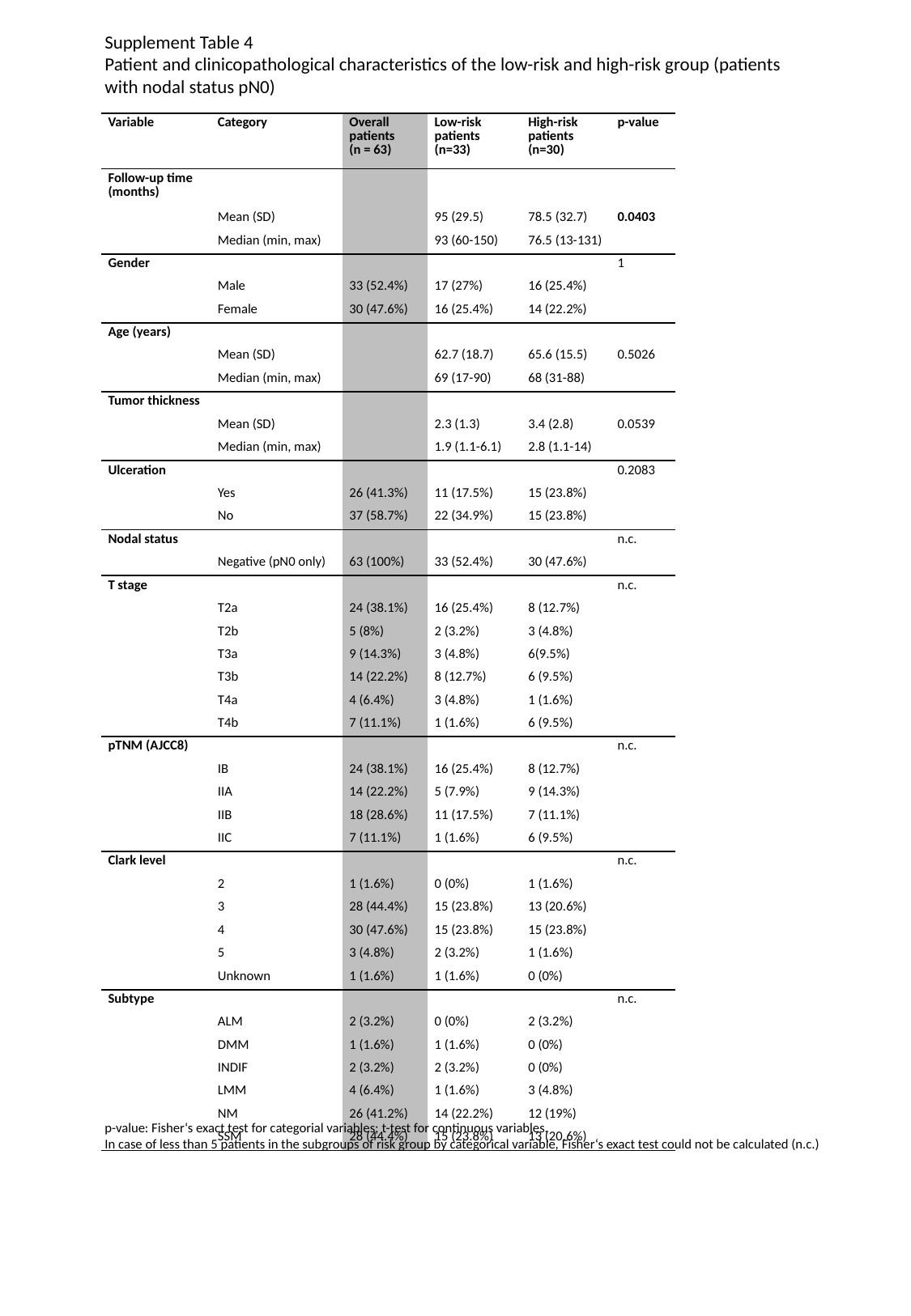

Supplement Table 4
Patient and clinicopathological characteristics of the low-risk and high-risk group (patients with nodal status pN0)
| Variable | Category | Overall patients (n = 63) | Low-risk patients (n=33) | High-risk patients (n=30) | p-value |
| --- | --- | --- | --- | --- | --- |
| Follow-up time (months) | | | | | |
| | Mean (SD) | | 95 (29.5) | 78.5 (32.7) | 0.0403 |
| | Median (min, max) | | 93 (60-150) | 76.5 (13-131) | |
| Gender | | | | | 1 |
| | Male | 33 (52.4%) | 17 (27%) | 16 (25.4%) | |
| | Female | 30 (47.6%) | 16 (25.4%) | 14 (22.2%) | |
| Age (years) | | | | | |
| | Mean (SD) | | 62.7 (18.7) | 65.6 (15.5) | 0.5026 |
| | Median (min, max) | | 69 (17-90) | 68 (31-88) | |
| Tumor thickness | | | | | |
| | Mean (SD) | | 2.3 (1.3) | 3.4 (2.8) | 0.0539 |
| | Median (min, max) | | 1.9 (1.1-6.1) | 2.8 (1.1-14) | |
| Ulceration | | | | | 0.2083 |
| | Yes | 26 (41.3%) | 11 (17.5%) | 15 (23.8%) | |
| | No | 37 (58.7%) | 22 (34.9%) | 15 (23.8%) | |
| Nodal status | | | | | n.c. |
| | Negative (pN0 only) | 63 (100%) | 33 (52.4%) | 30 (47.6%) | |
| T stage | | | | | n.c. |
| | T2a | 24 (38.1%) | 16 (25.4%) | 8 (12.7%) | |
| | T2b | 5 (8%) | 2 (3.2%) | 3 (4.8%) | |
| | T3a | 9 (14.3%) | 3 (4.8%) | 6(9.5%) | |
| | T3b | 14 (22.2%) | 8 (12.7%) | 6 (9.5%) | |
| | T4a | 4 (6.4%) | 3 (4.8%) | 1 (1.6%) | |
| | T4b | 7 (11.1%) | 1 (1.6%) | 6 (9.5%) | |
| pTNM (AJCC8) | | | | | n.c. |
| | IB | 24 (38.1%) | 16 (25.4%) | 8 (12.7%) | |
| | IIA | 14 (22.2%) | 5 (7.9%) | 9 (14.3%) | |
| | IIB | 18 (28.6%) | 11 (17.5%) | 7 (11.1%) | |
| | IIC | 7 (11.1%) | 1 (1.6%) | 6 (9.5%) | |
| Clark level | | | | | n.c. |
| | 2 | 1 (1.6%) | 0 (0%) | 1 (1.6%) | |
| | 3 | 28 (44.4%) | 15 (23.8%) | 13 (20.6%) | |
| | 4 | 30 (47.6%) | 15 (23.8%) | 15 (23.8%) | |
| | 5 | 3 (4.8%) | 2 (3.2%) | 1 (1.6%) | |
| | Unknown | 1 (1.6%) | 1 (1.6%) | 0 (0%) | |
| Subtype | | | | | n.c. |
| | ALM | 2 (3.2%) | 0 (0%) | 2 (3.2%) | |
| | DMM | 1 (1.6%) | 1 (1.6%) | 0 (0%) | |
| | INDIF | 2 (3.2%) | 2 (3.2%) | 0 (0%) | |
| | LMM | 4 (6.4%) | 1 (1.6%) | 3 (4.8%) | |
| | NM | 26 (41.2%) | 14 (22.2%) | 12 (19%) | |
| | SSM | 28 (44.4%) | 15 (23.8%) | 13 (20.6%) | |
p-value: Fisher‘s exact test for categorial variables; t-test for continuous variables.
In case of less than 5 patients in the subgroups of risk group by categorical variable, Fisher‘s exact test could not be calculated (n.c.)
