# Supplementary material for: Identifying High-Risk Tumors within AJCC Stage IB–III Melanomas Using a Seven-Marker Immunohistochemical Signature
Source: Cancers (Basel). 2021 Jun 10;13(12):2902. doi: 10.3390/cancers13122902 (PMC8229951; doi:10.3390/cancers13122902)
Supplement: Supplementary file 1 [file cancers-13-02902-s001.zip › cancers-1247549-supplementary/cancers-1247549-supplementary for XML/Supplement Table S5.pptx]

## Slide 1
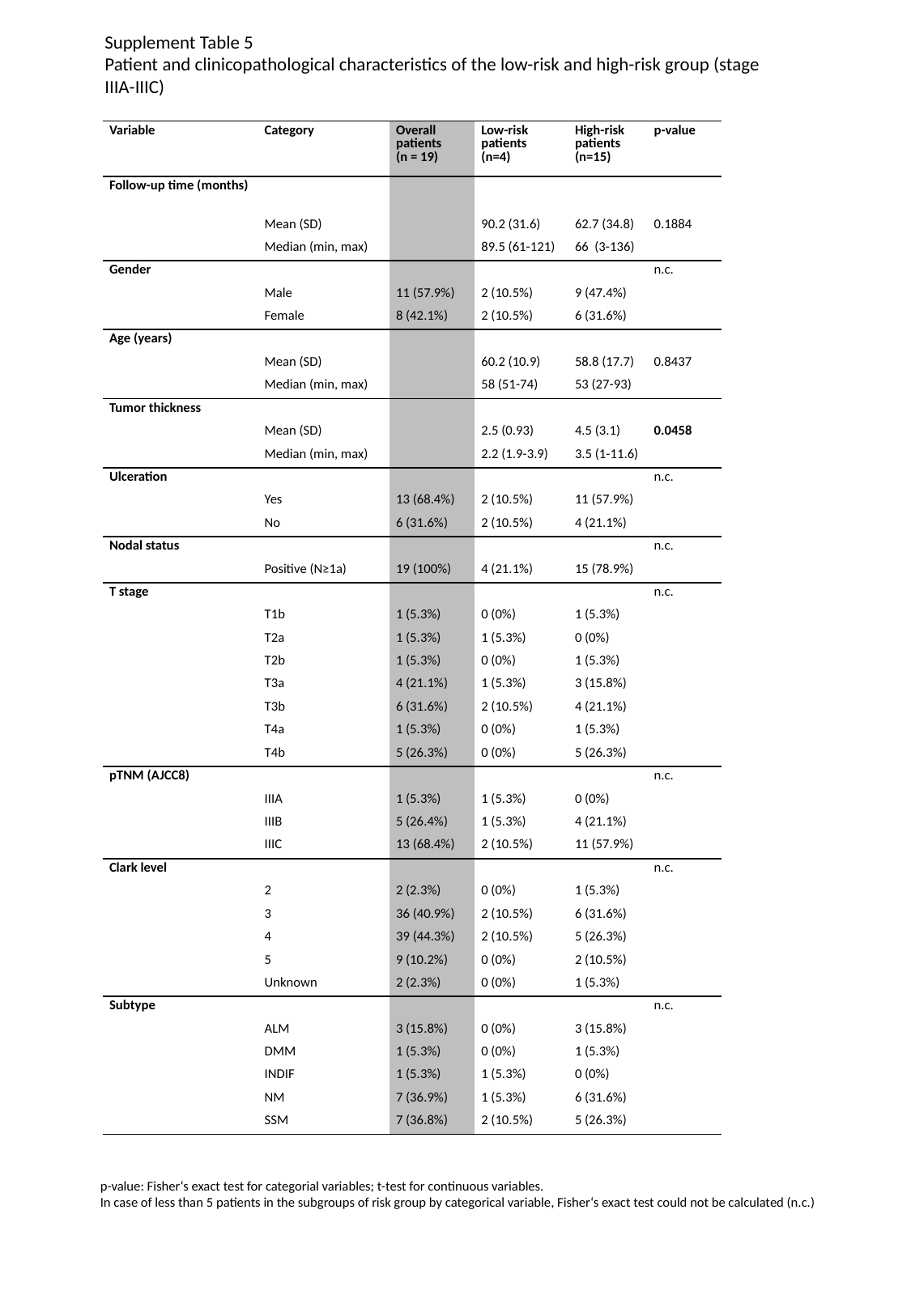

Supplement Table 5
Patient and clinicopathological characteristics of the low-risk and high-risk group (stage IIIA-IIIC)
| Variable | Category | Overall patients (n = 19) | Low-risk patients (n=4) | High-risk patients (n=15) | p-value |
| --- | --- | --- | --- | --- | --- |
| Follow-up time (months) | | | | | |
| | Mean (SD) | | 90.2 (31.6) | 62.7 (34.8) | 0.1884 |
| | Median (min, max) | | 89.5 (61-121) | 66 (3-136) | |
| Gender | | | | | n.c. |
| | Male | 11 (57.9%) | 2 (10.5%) | 9 (47.4%) | |
| | Female | 8 (42.1%) | 2 (10.5%) | 6 (31.6%) | |
| Age (years) | | | | | |
| | Mean (SD) | | 60.2 (10.9) | 58.8 (17.7) | 0.8437 |
| | Median (min, max) | | 58 (51-74) | 53 (27-93) | |
| Tumor thickness | | | | | |
| | Mean (SD) | | 2.5 (0.93) | 4.5 (3.1) | 0.0458 |
| | Median (min, max) | | 2.2 (1.9-3.9) | 3.5 (1-11.6) | |
| Ulceration | | | | | n.c. |
| | Yes | 13 (68.4%) | 2 (10.5%) | 11 (57.9%) | |
| | No | 6 (31.6%) | 2 (10.5%) | 4 (21.1%) | |
| Nodal status | | | | | n.c. |
| | Positive (N≥1a) | 19 (100%) | 4 (21.1%) | 15 (78.9%) | |
| T stage | | | | | n.c. |
| | T1b | 1 (5.3%) | 0 (0%) | 1 (5.3%) | |
| | T2a | 1 (5.3%) | 1 (5.3%) | 0 (0%) | |
| | T2b | 1 (5.3%) | 0 (0%) | 1 (5.3%) | |
| | T3a | 4 (21.1%) | 1 (5.3%) | 3 (15.8%) | |
| | T3b | 6 (31.6%) | 2 (10.5%) | 4 (21.1%) | |
| | T4a | 1 (5.3%) | 0 (0%) | 1 (5.3%) | |
| | T4b | 5 (26.3%) | 0 (0%) | 5 (26.3%) | |
| pTNM (AJCC8) | | | | | n.c. |
| | IIIA | 1 (5.3%) | 1 (5.3%) | 0 (0%) | |
| | IIIB | 5 (26.4%) | 1 (5.3%) | 4 (21.1%) | |
| | IIIC | 13 (68.4%) | 2 (10.5%) | 11 (57.9%) | |
| Clark level | | | | | n.c. |
| | 2 | 2 (2.3%) | 0 (0%) | 1 (5.3%) | |
| | 3 | 36 (40.9%) | 2 (10.5%) | 6 (31.6%) | |
| | 4 | 39 (44.3%) | 2 (10.5%) | 5 (26.3%) | |
| | 5 | 9 (10.2%) | 0 (0%) | 2 (10.5%) | |
| | Unknown | 2 (2.3%) | 0 (0%) | 1 (5.3%) | |
| Subtype | | | | | n.c. |
| | ALM | 3 (15.8%) | 0 (0%) | 3 (15.8%) | |
| | DMM | 1 (5.3%) | 0 (0%) | 1 (5.3%) | |
| | INDIF | 1 (5.3%) | 1 (5.3%) | 0 (0%) | |
| | NM | 7 (36.9%) | 1 (5.3%) | 6 (31.6%) | |
| | SSM | 7 (36.8%) | 2 (10.5%) | 5 (26.3%) | |
p-value: Fisher‘s exact test for categorial variables; t-test for continuous variables.
In case of less than 5 patients in the subgroups of risk group by categorical variable, Fisher‘s exact test could not be calculated (n.c.)
